# Inhibitory Effects of *Latilactobacillus curvatus* BYB3 Cell-Free Extract on Human Melanoma B16F10 Cells and Tumorigenic Mice

**DOI:** 10.4014/jmb.2309.09002

**Published:** 2023-10-30

**Authors:** Dingyun Li, Xing Wang, Dong-June Park, Dong Hun Lee, Sejong Oh

**Affiliations:** 1 Division of Animal Science, Chonnam National University, Gwangju 61186, Republic of Korea; 2 Department of Biochemistry Microbiology and Immunology, Wayne State University, Detroit, MI 48202, USA; 3 Korea Food Research Institute, Jeollabuk-do 55365, Republic of Korea; 4 Department of Biological Sciences, Chonnam National University, Gwangju 61186, Republic of Korea

**Keywords:** *Latilactobacillus curvatus*, melanoma B16F10 cell, cell migration, proliferation

## Abstract

*Latilactobacillus curvatus* BYB3 (BYB3) is a species of lactic acid bacteria, formerly named *Lactobacillus curvatus*, which is isolated from kimchi. In this study, the effect of BYB3, *Lactobacillus rhamnosus* GG, and *Lactobacillus acidophilus* GP1B strain extracts at various concentrations was examined on B16F10, a mouse melanoma cell line. Cell viability was examined via MTT assay, and the results indicated that compared to the other two probiotics, BYB3 significantly decreased the total percentages of viable cells. The effects of BYB3 on cell migration and proliferation in B16F10 cells were evaluated using wound healing mobility and proliferation assays, respectively; the results indicated that BYB3 inhibits cell migration and proliferation in a concentration-dependent manner. Using human dermal fibroblast cells to investigate BYB3 extract in vivo had no effect on skin-related cells. Nonetheless, the BYB3 extract inhibited tumor growth in a mouse model, as demonstrated by liver slices. Therefore, this suggests that using BYB3 extract to inhibit melanoma may be a novel approach.

## Introduction

Melanoma, a type of malignant skin cancer caused by melanocytes, continues to be a major public health concern due to its aggressive nature and limited therapeutic choices for advanced stages [1]. Despite advances in standard therapies such as surgery, chemotherapy, and radiation, melanoma's strong metastatic potential frequently results in treatment resistance and a poor prognosis for patients [2]. As a result, there is an urgent need to investigate novel and effective therapeutic options for melanoma care. Aside from UV exposure, major genetic abnormalities have been linked to melanoma [3].

The epithelial–mesenchymal transition (EMT) is a fundamental embryologic phenomenon that arises in wound healing and carcinogenesis [4]. However, cell culture was where EMT in cancer was initially described. Epithelial cells lose their intercellular adhesion and cell polarity during EMT and instead develop migratory and invasive characteristics that enable them to differentiate into mesenchymal cells. This transition and the emergence of new cellular characteristics are crucial for many developmental processes as well as the start of metastasis in the evolution of cancer. EMT facilitates cell invasion, which is necessary for the start of metastasis [5].

Lactic acid bacteria (LAB) derived from fermented food are most commonly used probiotics, which are well known for their substantial therapeutic effects in mammals [6]. LAB have a variety of physiological benefits, including as the improvement of hypercholesterolemia [7], hypertension [8], anticancer [9], and anti-inflammatory properties [10]. Additionally, in mice colitis models, the anti-inflammatory benefits of probiotics, such as *Latilactobacillus curvatus* BYB3 (BYB3), have been shown by Wang *et al*. in 2022 [11]. The effects of *L. curvatus* on various malignancies are currently being studied [12]. It is a significant species of LAB due to its potent inhibitory activity against cancerous (Caco-2 and HeLa) cells. Even though the impact of *L. curvatus* on melanoma cells is still unclear. Consequently, the purpose of the current investigation was to determine whether *L. curvatus*, in the form of a cell-free extract, has any impact on malignant melanoma cells. After treating melanoma cells, B16F10 (highly metastatic) with *L. curvatus* extract, the viability and molecular changes of the cells were analyzed.

The mechanisms of action of BYB3 extract will be clarified by an understanding of how it affects cell proliferation, migration, and gene expression, which may lead to the identification of novel targets for the treatment of melanoma. Additionally, by examining its effect on skin-related cells, its selectivity for cancer cells will be confirmed, allaying worries about unintended side effects. In general, this study holds promise in establishing a foundation for the creation of innovative probiotic-centered treatments for melanoma, with potential ramifications extending to the wider domain of cancer therapy. By elucidating the anticancer potential of BYB3 extract, this study aims to contribute to the ongoing efforts to combat this devastating disease and improve patient outcomes.

## Materials and Methods

### Materials

Fetal bovine serum, 3-(4,5-dimethylthiazol-2-yl)-2,5-diphenyltetrazolium bromide, and antibiotic-antimycotic solution (P/S) were purchased from HyClone (USA), Sigma-Aldrich (USA), and Gibco (USA), respectively. The anti-CDH1 Polyclonal Antibody (Cat No. K003568P), anti-CADH2 Polyclonal Antibody (Cat No. K003569P), anti-Vimentin Polyclonal Antibody (Cat No. K108978P) and β-actin antibody (Cat No. K200058M) were purchased from Solarbio China while secondary R-antibody (A11036) was purchased from Invitrogen (USA). Westar supernova (cad.XLS3,0100) was purchased from Cyanagen, Italy.

### Cell Culture

B16F10 cells were provided by the Korean Cell Line Bank (Republic of Korea). The cells were cultured in Dulbecco’s Modified Eagle Medium (DMEM-high-glucose medium; Welgene, Republic of Korea) supplemented with 10% fetal bovine serum (Welgene) and 1% antibiotic-antimycotic solution (Gibco) under conditions of 5%CO_2_ and 37ºC. The medium was replaced twice or thrice in a week. Cells were seeded in 96-well or 6-well plates. The final volumes of culture were 100 μl/well in 96-well plate and 2 ml/well in 6-well plates, respectively.

### Preparation of LAB Extract

The BYB3, *Lactobacillus acidophilus* GP1B (GP1B), and *Lacticaseibacillus rhamnosus* GG (LGG) strains were extracted/isolated from kimchi, swine intestine, and yogurt, respectively. The three strains were cultured in sterilized MRS broth (Difco, USA) at 37°C for 24 h. The cultured strains were centrifuged at 10,000 ×*g* for 15 min at 4°C for harvest. The strain pellet was washed with distilled water (DW) thrice to remove the MRS medium. After the washing process, the strain pellet was resuspended with DW and sonicated for 30 min on ice. The sonicated strains were centrifuged at 12,000 ×*g* for 15 min at 4°C. The supernatant was filtered using a 0.45 μm filter (Sartorius AG, Germany) and freeze-dried for 72 h. The extract was dissolved at 50 mg/ml in phosphate-buffered saline (PBS) as standard solution and subsequent experiments were conducted using a 0.45 μm filter.

### Crystal Violet Staining Assay

Crystal violet staining assay was used to evaluate whether BYB3 affects cell proliferation. Briefly, cells were seeded in 6-well plates with DMEM at a density of 6.25 × 10^5^ cells per well and then incubated with BYB3, GP1B, and LGG strains at different concentrations (50, 100, and 150 μg/ml) or dimethyl sulfoxide (DMSO) as control for 3 days. Cells were washed twice with PBS, and fixed with 4% paraformaldehyde; subsequently, they were stained with saturated crystal violet solution for 20 min at room temperature. Following this, crystal violet solution was removed, and resulting cells were washed twice with tap water, Further, the samples were photographed using a microscope.

### Cell Viability Assay

Viability of B16F10 cells was determined by performing the 3-(4,5-dimethylthiazol-2-yl)-2,5-diphenyltetrazolium bromide (MTT) assay. B16F10 cells were seeded at a density of 2 × 10^3^ cells per well in 96-well plates and allowed to adhere for 24 h. The cells were treated with serum-free DMEM of the three strain extracts of various concentrations and incubated for 72 h. Then, the DMEM was discarded with the extract, followed by the addition of the mixture of 100 μl extract with 5 mg/ml of MTT solution dissolved in phosphate buffer (pH = 6.8) and subsequent incubation again for 4 h. The MTT solution was then removed and 100 μl dimethyl sulfoxide (Sigma-Aldrich) was added. Absorbance was measured at 490 nm using a microplate reader of Synergy HTX (USA).

### Cell Migration Assay

B16F10 cells were seeded in 12-well plates and cultured until they reached approximately 70% confluence. Subsequently, the B16F10 cells were treated with cyclophosphamide monohydrate (Sigma-Aldrich) for 3 h before the cell migration assay to exclude the effect of proliferation. A scratch was drawn in the center of the well using a 200 μl pipette tip. Each well was washed thrice with PBS to remove cyclophosphamide monohydrate and dead cells. B16F10 cells were then treated with various concentrations of the BYB3 extract.

### Immunoblotting

For immunoblotting, B16F10 cells were lysed by PRO-PREP protein extraction solution (iNtRON Biotechnology). Briefly, 5 × 10^6^ cells were immersed in 400 μl of the PRO-PREP solution and homogenized in ice for 10–20 min. The mixture of cell lysates was then centrifuged at 13,000 rpm for 15 min. The supernatant was collected, and the protein concentration was determined by the Pierce BCA Protein Assay Kit (Thermo Fisher Scientific). The polypeptides were separated on 4–20% SDS–PAGE gradient gels and then transferred to a polyvinylidene difluoride (PVDF) membrane (Bio-Ras Laboratories). The member was blocked with 5% skim milk (Difco Laboratories) and underwent overnight antibody incubation Primary Abs against the following proteins were used: human/mouse NLRP3 (1:1000, AP-20B-0014, AdipoGen), human NCOA6 (1:1000, A300-411A, Bethyl Laboratories), human/mouse ASC (1:1000, sc-514414, Santa Cruz Biotechnology; AG-25B-0006, AdipoGen), IL-1β (1:500, AF-401-NA, R&D Systems), mouse cleaved caspase-1 (1:1000, AG-20B-0042, AdipoGen), FLAG (1:1000, sc-166355, Santa Cruz Biotechnology), Myc (1:1000, ab13836, Abcam), GAPDH (1:1000, sc-25778, Sigma).

### Mice Assay Xenograft Tumor Growth

9-week-old of age female BALB/c mice were acclimated for 7 days after arrived and hosed in cages according to their body weight. All mice were maintained under specific pathogen-free conditions. A few 4 × 10^6^ B16f10 cells with injected subcutaneously to the flank of 10-week-old BALC/c female mice. Mice were then monitored weekly for tumor formation. All the animal experiments were conducted in accordance with the protocol approved by Chonnam National University Institutional Animal Care and Use Committee (CNU IACUC-YB-2021-76).

### Mice Experimental Design

The animals (*n* = 18) used in the experiment were divided into three groups (Fig. 3C):

Group 1- Control: the group were intraperitoneally injected into the BALB/c mice in 200 μl phosphate buffer saline.

Group 2-Melanoma: the group injection of B16F10 cells (5 × 10^5^) were subcutaneously inoculated into the BALB/c mice and were intraperitoneally 200 μl phosphate buffer saline.

Group 3-Melanoma+ BYB3 extract: the group injection of B16F10 cells (5 × 10^5^) were subcutaneously inoculated into the BALB/c mice and were intraperitoneally 200 μl phosphate buffer saline with BYB3 extract 500 mg/kg.

### Histological Assessment

Liver collected were washed several times with sterile PBS until the cleared. A part of the liver tissue was then fixed with 10% phosphate-buffered formalin overnight. After fixation, the tissue samples were dehydrated using a low-to-high-concentration ethanol series. The tissue was paraffin embedded. The paraffin blocks were sectioned (5 μm thick) and stained with hematoxylin-eosin for histological assessment. The histology images were assessed under light microscope with 10× magnification and were quantified using Images were assessed as reported [13].

## Results

### BYB3 Extract Most Obviously Affected the Growth and Viability of B16f10 Cells

**Fig. 1:**
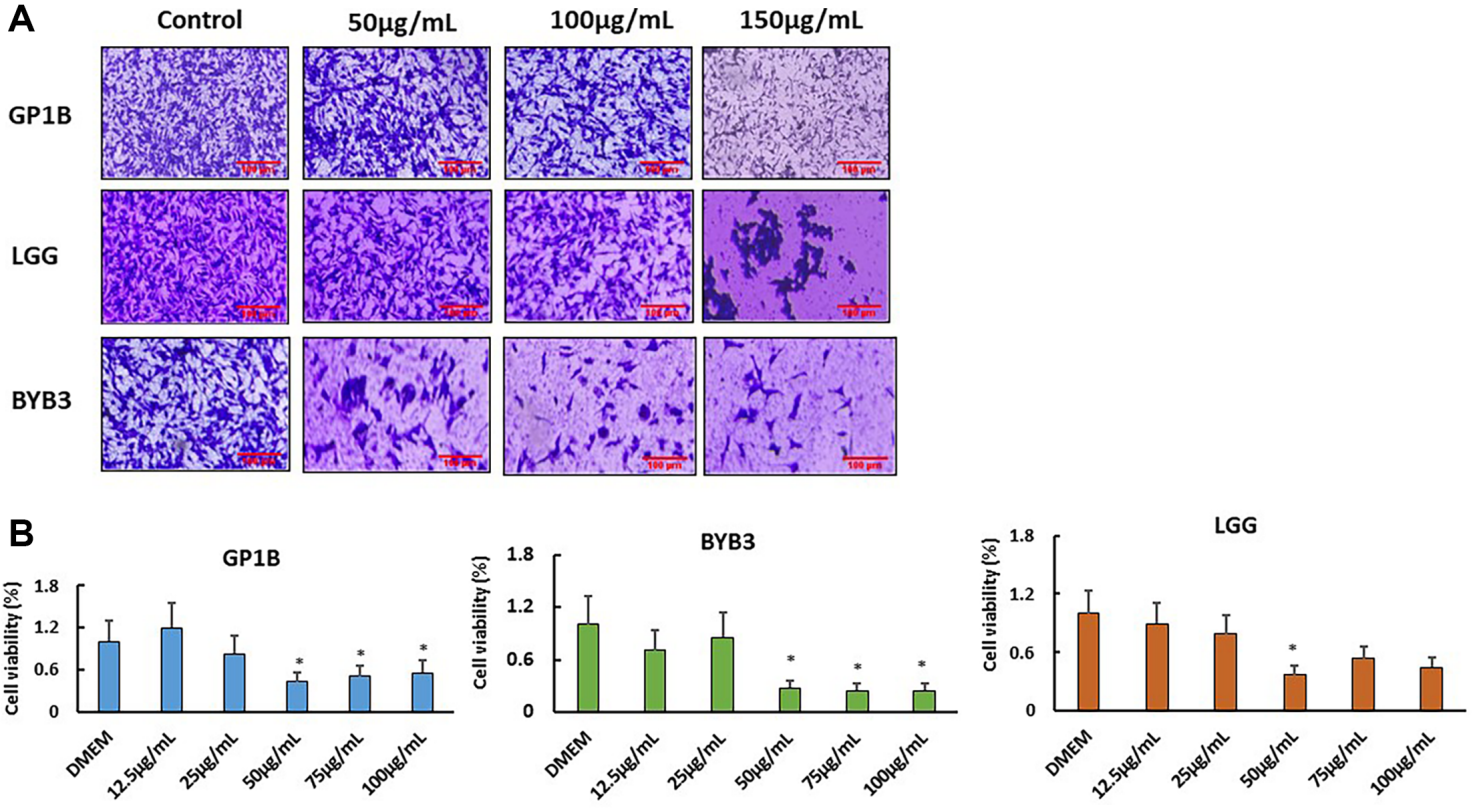
Effects of BYB3, GP1B, and LGG extracts on the proliferation of B16F10 cells. (**A**) The cells were first stained with crystal violet solution. Subsequent examination showed that the three strain extracts were not affected by the malignancy in the B16F10 cells; however, the proliferation of BYB3-treated cell was inhibited more than that of LGG and GP1B treated cells. (**B**) Viability began to decrease in a dose-dependent manner in the B16F10 cells as the concentration of the three strain extracts decreased from 75 μg/ml to lower concentrations than in BYB3. Statistically significant differences are shown. (*p < 0.05, **p < 0.01).

Using 50, 100, 150 μg/ml concentrations of BYB3, GP1B, and LGG extracts on melanoma cells. After 72 h of treatment, the crystal violet solution was used to observe the growth and appearance of cells in the wells (Fig. 1A), whereas that of the B16f10 cells was dose-dependently inhibited in all treated groups. The growth of BYB3 strain was significantly decreased from 50 μg/ml treated, and compared with GP1B and LGG, the decrease was more obvious at 100 μg/ml treated group. Further, the quantitative analysis of the 3 strains treated melanoma cells was conducted after 72 h by MTT viability assay (Fig. 1B).

### BYB3 Extract Inhibited the Migration of B16F10 Cells

**Fig. 2:**
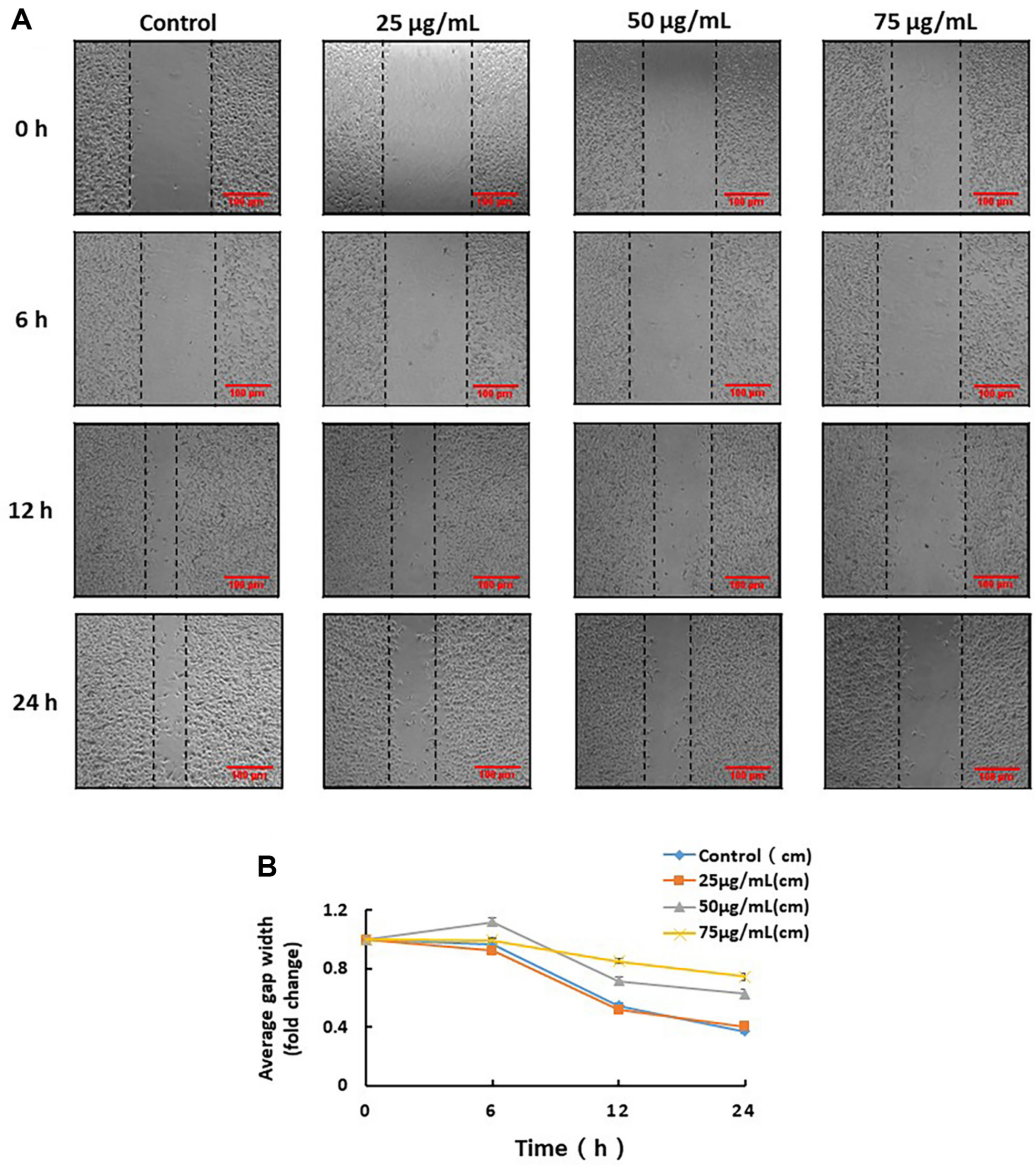
Analysis of cell migration ability subsequent to the treatment of B16F10 cell with BYB3 extract. The cells were cultured with the BYB3 extract subsequent to scratching, using a 200 μl pipette tip. (**A**) The B16F10 cells were observed every 6 h subsequent to scratching and treatment with BYB3 extract. (**B**) The average gap width was quantified. (A and B; *p < 0.05 versus the control group at 6, 12, and 24 h. Scale bar = 100 μm.

To further evaluate the anti-migratory effect of the BYB3 extract on B16f10 cells, the gap width was measured following the treatment of the cells with the BYB3 extract. We observed that migration was reduced in the BYB3 extract-treated groups, compared to that in the control group, in a dose-dependent manner (Fig. 2). Further, we adjusted the concentration of the BYB3 extract (25, 50, and 75 μg/ml) and performed a scratch assay to assess the degree of migration; we found that treatment with 75 μg/ml of BYB3 extract for 24 h significantly decreased cell migration, and the gap width of the was rather widened significantly decreased (Fig. 2A). The migration of cells was quantified and analyzed by measuring the average gap width (Fig. 2B).

### BYB3 Extract Changed the Protein Expression of Genes associated with Metastasis

**Fig. 3:**
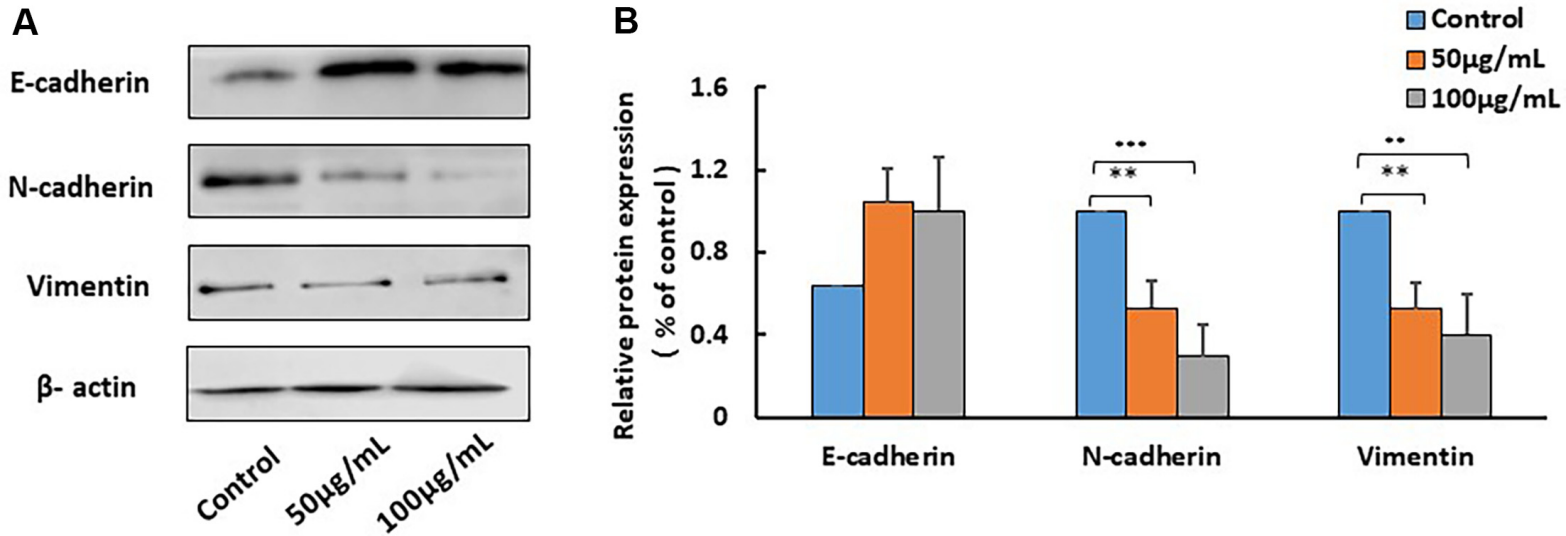
Variations in gene expression levels of migration-related genes subsequent to the treatment of B16F10 cells with BYB3-extract. (**A**) The protein expression levels of N-cadherin and Vimentin slightly decreased following the treatment with BYB3 extract. The expression of E-cadherin was upregulated in B16F10 cells. (**B**) Values of protein were normalized against actin as control. Statistically significant differences are shown. (*p < 0.05, **p < 0.01).

The molecular changes in mesenchymal gene expression after the treatment were detected by western blotting. The concentration of BYB3-extract was determined as mentioned above (50 and 100 μg/ml). The protein expression of mesenchymal cell markers including N-cadherin and Vimentin was decreased with increasing BYB3-extract concentration. However, the expression of Vimentin was observed to be only reduced in the protein level of B16F10 cells (Fig. 3).

### BYB3 Extract not Affected the Skin-Related Cell

**Fig. 4:**
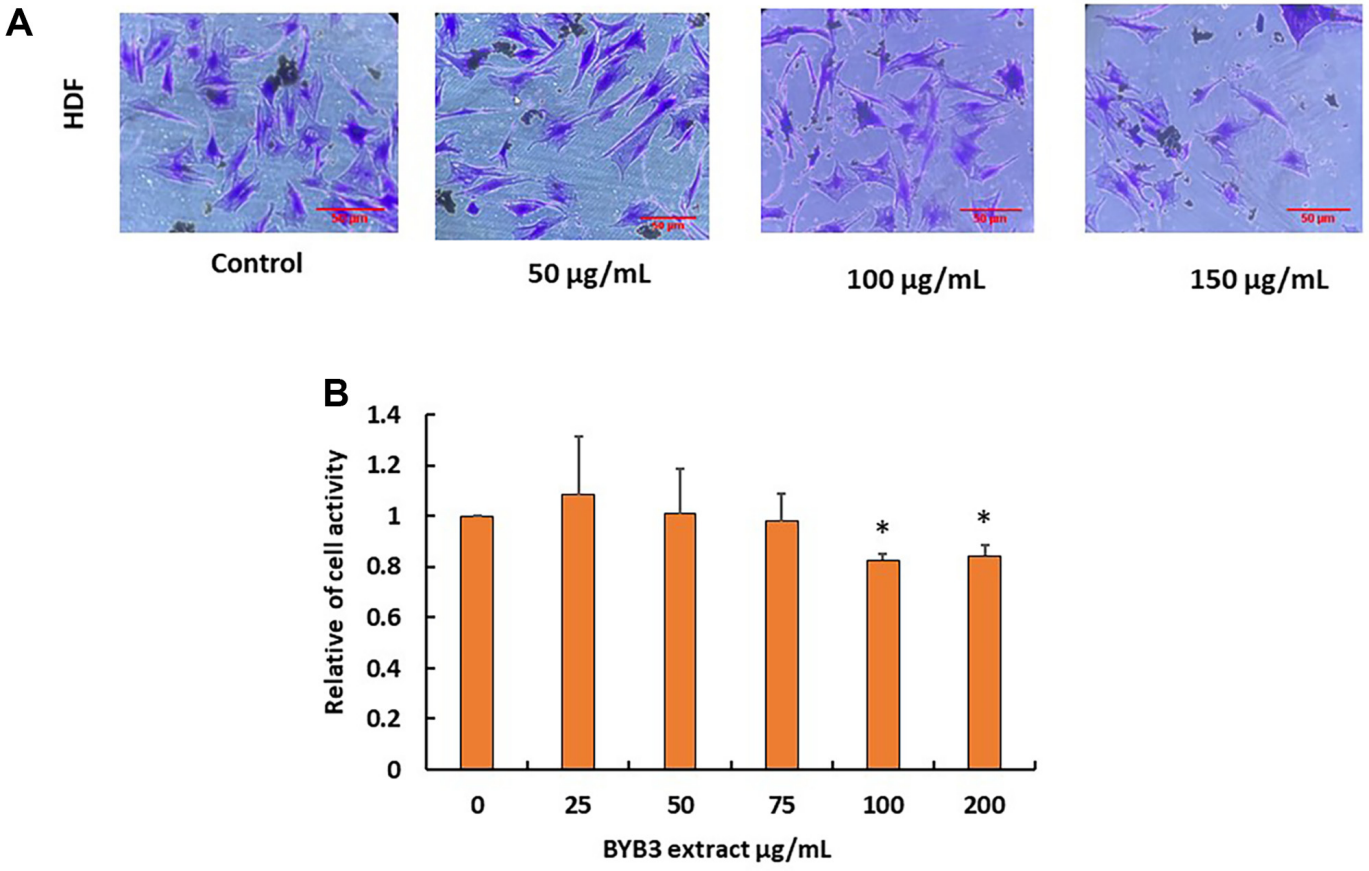
Effect of the BYB3 extract on the growth of human dermal fibroblast (HDF). (**A**) Effect of 72 h treatment of the BYB3 extract on the growth of human dermal fibroblast (HDF) after 72 h. The cells were stained with crystal violet solution. (**B**) Viability began to decrease from high concentration 100 μg/ml as dose-dependent manner in HDF. (**C**) The schedule of mouse treatment.

To investigate the effects of the BYB3 extract on human skin-related cells, human dermal fibroblasts (HDF) was treated with 50, 100, and 150 μg/ml BYB3 extracts. After 72 h of treatment, the growth and appearance of the cells in the well were observed using the crystal violet solution (Fig. 4A). The growth of HDF was decreased in the 100 μg/ml treated group, for quantitative analysis the viability of cells was measured after 72 h of treatment by MTT viability assay. The growth-inhibitory effect of the BYB3 extract was also confirmed by the in vivo experiment. The process of mice assay showed in the (Fig. 4C) divided three groups. Group 1 was intraperitoneally injected into 200 μl phosphate buffer saline from 12nd days, group 2 was acclimated later then injection of B16F10 cells (5 × 10^5^) were subcutaneously in 7^th^ day, and intraperitoneally 200 μl phosphate buffer saline from 12nd days every 2 days once. Group3 and the second group were all the same except for intraperitoneally injection 200 μl BYB3 extract.

### BYB3 Extract Inhibited Tumor Growth in vivo

**Fig. 5:**
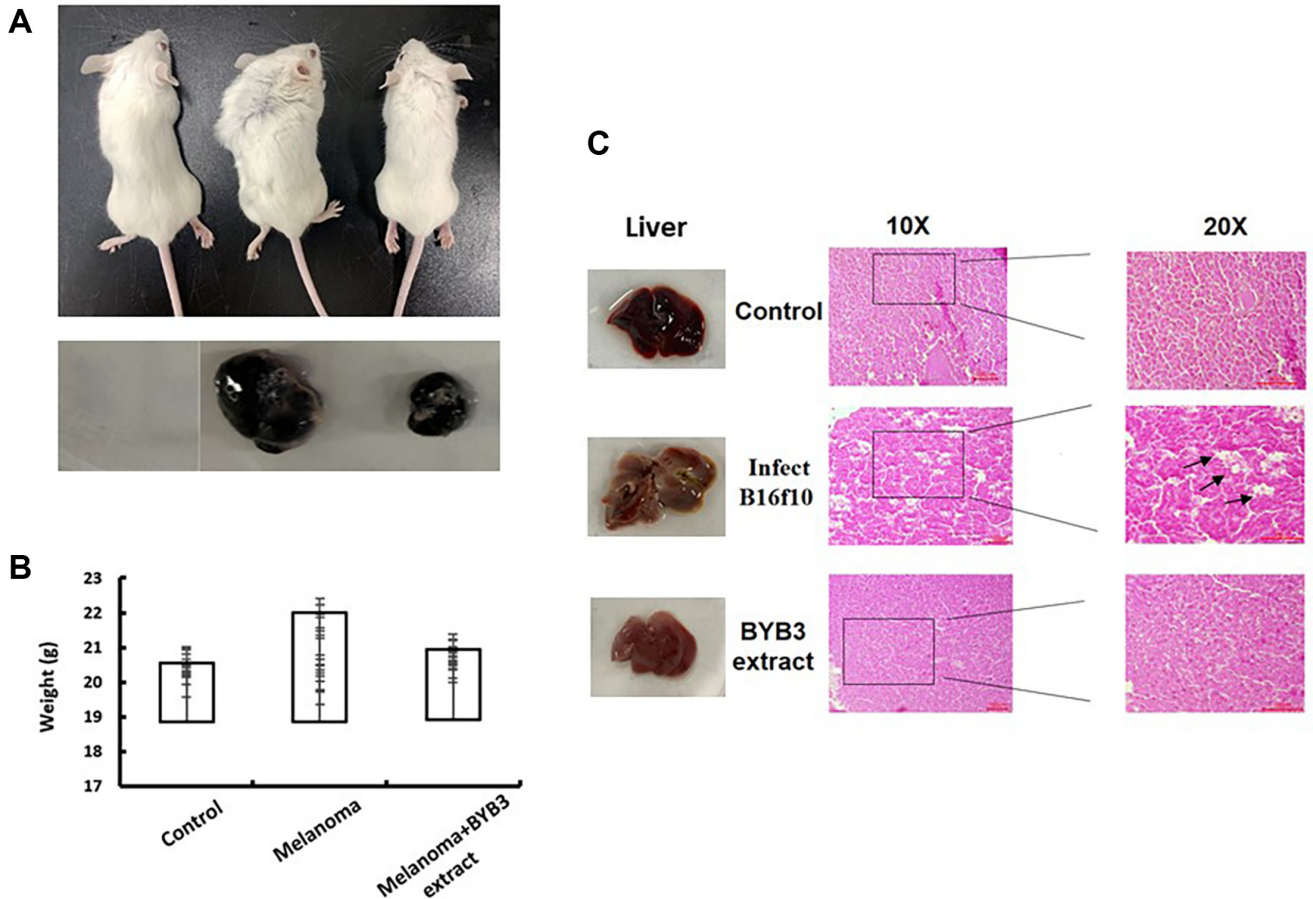
In vivo study of anticancer effect of the BYB3 extract. (**A**) Subcutaneous tumor size was documented after with PBS, B16F10+PBS, B16F10+BYB3 extract. Images of each tumor after excision from mice. (**B**) The weight of the mice was quantified. (**C**) Images of each liver after excision from mice. Liver hematoxylin–eosin staining in different groups. Each bar represents the mean ± SEM for groups of six. Scale bar = 50 μm for 10× and 20× magnification.

For better mimicking the in vivo environment for cancer therapy, the solid tumor mice model was developed to assess the antitumor performance of the BYB3 extract. Therefore, for further investigation of the anti-melanoma effect of BYB3 extract in vivo, mouse tumor models were established by subcutaneously inoculating B16F10 cells into two groups of mice, which would be dealt with phosphate buffer saline (group1), melanoma tumor (group2), BYB3 extract (group3), respectively. The photos of the cancer were showed in Fig. 5A. The growth-inhibitory effect of the BYB3 extract was also confirmed by the in vivo experiment. In the BYB3 extract treated group, the tumors induced by subcutaneously injecting BYB3 were evidently smaller than those of the melanoma group. The weight of the mice was quantified (Fig. 5B) of 500mg/kg BYB3 extract significantly decreased the melanoma tumor group. Histopathological results of liver tissue showed that group 2 mice developed a considerably high level of hepatic steatosis (black arrow). On the contrary, the group 3 with subcutaneously of BYB3 extract and much smaller size of group 2 could be observed. Taken together, these results supported that BYB3 extract could improve melanoma migration liver injury mice (Fig. 5C).

## Discussion

The present study aimed to investigate the potential anticancer properties of BYB3 extract in the context of melanoma treatment. Our research demonstrates substantial effects on B16F10 melanoma cells both in vitro and in vivo, suggesting the BYB3 extract has considerable therapeutic potential for the treatment of melanoma.

The in vitro results showed that BYB3 extract had a dose-dependent inhibitory effect on the proliferation and viability of B16F10 cells. At the lowest concentration of 50 g/ml, this growth inhibition was noticeable, and at 100 g/ml, it was more apparent. These results are consistent with earlier research that found probiotics and their extracts have anticancer action against a number of cancer cell lines, including melanoma [14, 15]. The observed restriction of cell development raises the possibility that BYB3 extract may obstruct crucial cellular functions, impairing proliferation and eventually causing cell death in B16F10 cells.

In addition to growth inhibition, B16F10 cell migration was significantly impacted by BYB3 extract. The results of the scratch assay showed dose-dependent reductions in cell movement. Cell migration is a critical aspect of cancer metastasis, and targeting this process represents a potential strategy for limiting cancer progression. The ability of BYB3 extract to regulate the expression of genes linked to metastasis may be the cause of its anti-migratory effects, as shown by alterations in the mesenchymal cell markers N-cadherin and Vimentin. The downregulation of these markers suggests that treated cells may become less aggressive and more epithelial [16]. It is interesting that only the protein level of Vimentin was down-regulated, suggesting that this mesenchymal signal may be subject to post-transcriptional control.

Importantly, according to the results of our study, BYB3 extract did not harm human dermal fibroblasts, demonstrating some degree of selectivity in its cytotoxic actions on melanoma cells. To reduce negative effects on healthy cells and tissues, anticancer drugs must be selective in order to be effective. Our in vitro and in vivo tests revealed that the extract had no discernible effect on cells associated to the skin, indicating its potential as a secure therapeutic choice for the treatment of melanoma.

In the present in vivo investigation, the administration of BYB3 extract demonstrated a substantial inhibition of tumor growth in a murine model of melanoma. The observed decrease in tumor growth in the group treated with the BYB3 extract provides additional evidence to support the possible therapeutic effectiveness of the extract. Additionally, the histological analysis demonstrated a reduction in hepatic steatosis within the experimental group, indicating a potentially advantageous effect on liver functionality.

The findings of this study are consistent with earlier studies that have shown probiotics and their extracts to have anticancer effects. The modulation of the tumor microenvironment, augmentation of the host immune response, and initiation of apoptosis in cancer cells have been demonstrated as effects of probiotics [17, 18]. This study contributes to the growing body of research by explicitly identifying BYB3 extract as a possible therapeutic agent for the treatment of melanoma.

Nevertheless, it is imperative to recognize specific constraints within this research. Initially, it is important to note that while our experimental results conducted inside a living organism demonstrate encouraging outcomes in terms of inhibiting tumor growth, additional research is necessary to comprehensively understand the precise pathways via which BYB3 extract operates. Furthermore, it is imperative to conduct further studies in both preclinical and clinical environments to validate the safety and effectiveness of BYB3 extract. Further research is required to identify the precise bioactive components that cause the observed effects, allowing for the creation of more specialized and effective treatment formulations.

In conclusion, this research offers strong evidence supporting the potential of BYB3 extract as an anticancer agent against melanoma cells. The extract demonstrated significant inhibitory effects on cell growth and migration, as well as the modulation of gene expression associated with metastasis. Furthermore, the study demonstrated that there were no adverse impacts observed on cells associated with healthy skin. The significance of investigating probiotics as potential adjuvant therapy for the treatment of cancer is highlighted by these findings. Additional investigation and rigorous clinical studies are vital in order to substantiate the therapeutic effectiveness and safety of the BYB3 extract. These efforts are crucial for the eventual translation of the extract into a practical and efficient therapy alternative for individuals suffering from melanoma.
